# Supplementary material for: A UMLS-based spell checker for natural language processing in vaccine safety
Source: BMC Med Inform Decis Mak. 2007 Feb 12;7:3. doi: 10.1186/1472-6947-7-3 (PMC1805499; doi:10.1186/1472-6947-7-3)
Supplement: Additional file 13 — RAP application directory. Sets up directory for RAP (RDF application for PHP) source code [file 1472-6947-7-3-S13.gz › rap/doc/phpdoc/classtrees_dataset.html]

xml version="1.0" encoding="iso-8859-1"?


## Root class Dataset

- Dataset
  - DatasetDb
  - DatasetMem

## Root class IteratorAllGraphsDb

- IteratorAllGraphsDb

## Root class IteratorAllGraphsMem

- IteratorAllGraphsMem

## Root class IteratorFindQuadsDb

- IteratorFindQuadsDb

## Root class IteratorFindQuadsMem

- IteratorFindQuadsMem

## Root class NamedGraphDb

- DbModel **(Different package)**
  - NamedGraphDb

## Root class NamedGraphMem

- MemModel **(Different package)**
  - NamedGraphMem

## Root class Quad

- Quad

## Root class TriXParser

- TriXParser

## Root class TriXSerializer

- TriXSerializer

Documentation generated on Thu, 7 Jul 2005 13:41:07 +0200 by phpDocumentor 1.3.0RC3
